# Insulin-like growth factor 1 receptor expression correlates with programmed death ligand 1 expression and poor survival in non-small cell lung cancer

**DOI:** 10.1371/journal.pone.0297397

**Published:** 2024-10-04

**Authors:** Hiroaki Nagamine, Masakazu Yashiro, Megumi Mizutani, Akira Sugimoto, Yoshiya Matsumoto, Yoko Tani, Kenji Sawa, Hiroyasu Kaneda, Kazuhiro Yamada, Tetsuya Watanabe, Kazuhisa Asai, Satoshi Suzuki, Tomoya Kawaguchi

**Affiliations:** 1 Department of Respiratory Medicine, Graduate School of Medicine, Osaka City University, Osaka, Japan; 2 Department of Respiratory Medicine, Graduate School of Medicine, Osaka Metropolitan University, Osaka, Japan; 3 Molecular Oncology and Therapeutics, Graduate School of Medicine, Osaka Metropolitan University, Osaka, Japan; 4 Department of Clinical Oncology, Graduate School of Medicine, Osaka Metropolitan University, Osaka, Japan; 5 Department of Thoracic Surgery, Graduate School of Medicine, Osaka Metropolitan University, Osaka, Japan; National Taiwan University College of Medicine, TAIWAN

## Abstract

The insulin-like growth factor 1 receptor (IGF1R) has been associated with growth and metastasis in various cancers. However, its role in postoperative recurrence and prognosis in lung cancer lacks clear consensus. Therefore, this study aimed to investigate the potential relationship between IGF1R and postoperative recurrence as well as long-term survival in a large cohort. Additionally, we assessed the relationship between IGF1R and programmed death ligand 1 (PD-L1) expression. Our study encompassed 782 patients with non-small cell lung cancer (NSCLC). Immunostaining of surgical specimens was performed to evaluate IGF1R and PD-L1 expression. Among the patients, 279 (35.8%) showed positive IGF1R expression, with significantly worse relapse-free survival (RFS) and overall survival (OS). Notably, no significant differences in RFS and OS were observed between IGF1R-positive and -negative groups in stages 2 and 3. However, in the early stages (0–1), the positive group displayed significantly worse RFS and OS. In addition, PD-L1 expression was detected in 100 (12.8%) patients, with a significant predominance in the IGF1R-positive. IGF1R may serve as a prognostic indicator and a guide for perioperative treatment strategies in early-stage lung cancer. In conclusion, our findings underscore an association between IGF1R expression and poor survival and PD-L1 expression in NSCLC.

## Introduction

The insulin-like growth factor 1 receptor (IGF1R) is expressed in various cancers [1–3] and is associated with cancer growth [4], metastasis [5, 6], and malignancy [7, 8]. In lung cancer, particularly non-small cell lung cancer (NSCLC), IGF1R expression is associated with carcinogenesis and proliferation [9, 10]. Nevertheless, the association between IGF1R expression and postoperative prognosis as well as recurrence in lung cancer has been inconsistent [11–14], lacking a definitive consensus.

In recent years, the expression of programmed death ligand 1 (PD-L1) has attracted attention as a predictor of immune checkpoint inhibitor (ICI) efficacy in the treatment of lung cancer [15]. Although the expression of IGF1R and PD-L1 has been implicated in head and neck squamous cell carcinoma [16], their role in lung cancer remains unknown. The development of lung cancers, particularly squamous cell carcinoma (SCC), has been associated with smoking [17]. Moreover, smoking has been known to be involved in tumor-induced IGF1R expression [10]. However, it remains unclear whether IGF1R expression is associated with smoking and PD-L1 expression in actual clinical practice and whether it is involved in postoperative recurrence and survival in lung cancer. Therefore, this study aimed to investigate the potential associations between IGF1R expression, PD-L1 expression, smoking history, postoperative recurrence, and survival in lung cancer. To our knowledge, this is the first study to comprehensively evaluate the relationship between IGF1R and these critical factors using long-term observations on a large scale. We anticipate that our findings will contribute to the ongoing efforts to optimize lung cancer management, leading to more effective and personalized therapeutic interventions in the realm of precision medicine.

## Materials and methods

### Patient and data collection

A total of 782 patients with NSCLC who underwent surgery at the Osaka Metropolitan University Hospital between January 2010 and December 2019 were included in this study. Cancer stage for all patients was assessed according to the Union for International Cancer Control, 8th edition. Relapse-free survival (RFS) was calculated from the date of surgery to the date of first recurrence or death from any cause. Overall survival (OS) was calculated from the date of surgery to the date of death from any cause. Our observations were conducted retrospectively, with August 31, 2023, as the data cutoff date, spanning a maximum of 10 years. At the cut-off date or 10 years after the start of observation, cases with no events were terminated from observation. Between September 1, 2023, and October 31, 2023, we accessed medical records for the collection of information, including personally identifiable details, related to registered patients. This study was approved by the Osaka City University Ethics Committee (reference number 2019–006). Informed consent was obtained from each patient. This study was conducted according to the principles of the Declaration of Helsinki.

### Immunohistochemistry

Immunohistochemical staining was performed on paraffin-embedded sections of primary surgically resected specimens obtained from patients with NSCLC. Slides were deparaffinized using xylene and rehydrated with gradually decreasing concentrations of ethyl alcohol. The slides were then incubated with methanol containing 3% hydrogen peroxide at room temperature for 15 min to eliminate endogenous peroxidase activity. Subsequently, the slides were heated in an autoclave at 105°C for 10 min in Target Retrieval Solution (Dako, Santa Clara, CA, USA). Following this, the slides were blocked with 10% normal mouse serum for 10 min at room temperature and incubated overnight at 4°C with the anti-IGF1R antibody (NB110-87052, 1:200; Novus Biologicals LLC, Centennial, CO, USA). Subsequently, the samples were incubated with a biotinylated secondary antibody for 10 min at room temperature, followed by treatment with streptavidin-peroxidase reagent for 5 min at room temperature, incubation in diaminobenzidine for 2 min at room temperature, and counterstaining with 100% Mayer’s hematoxylin for 40 s at room temperature. For PD-L1 staining, sections were blocked with 10% normal rabbit serum, and an anti-PD-L1 antibody (ab205921, 1:150, Abcam, Cambridge, UK) was used as the primary antibody, followed by reaction with diaminobenzidine for 7 min.

### Statistical analysis

The χ2 test was employed to evaluate the significance of differences in patient characteristics between the IGF1R-positive and IGF1R-negative groups. Multiple logistic regression analysis was utilized to analyze the relationship between IGF1R and PD-L1 expression, incorporating age, sex, smoking history, Eastern Cooperative Oncology Group Performance Status (ECOG PS), histology, pStage, pleural invasion, lymphatic invasion, and vascular invasion as factors. The Mann–Whitney U test evaluated differences in smoking index or maximum tumor diameter between the IGF1R-positive and negative groups. Survival curves were generated using Kaplan–Meier method, and the log-rank test was used to compare cumulative survival durations in the patient groups. In addition, the Cox proportional hazards model was used to compute univariable and multivariable hazard ratios for the study parameters. Analyses were performed using EZR in R Commander version 1.54 (Saitama Medical Center, Jichi Medical University, Saitama, Japan). In all tests, *p*<0.05 was considered to indicate statistical significance.

## Results

### Patient characteristics and the association of IGF1R expression with smoking history and tumor size

**Fig 1:**
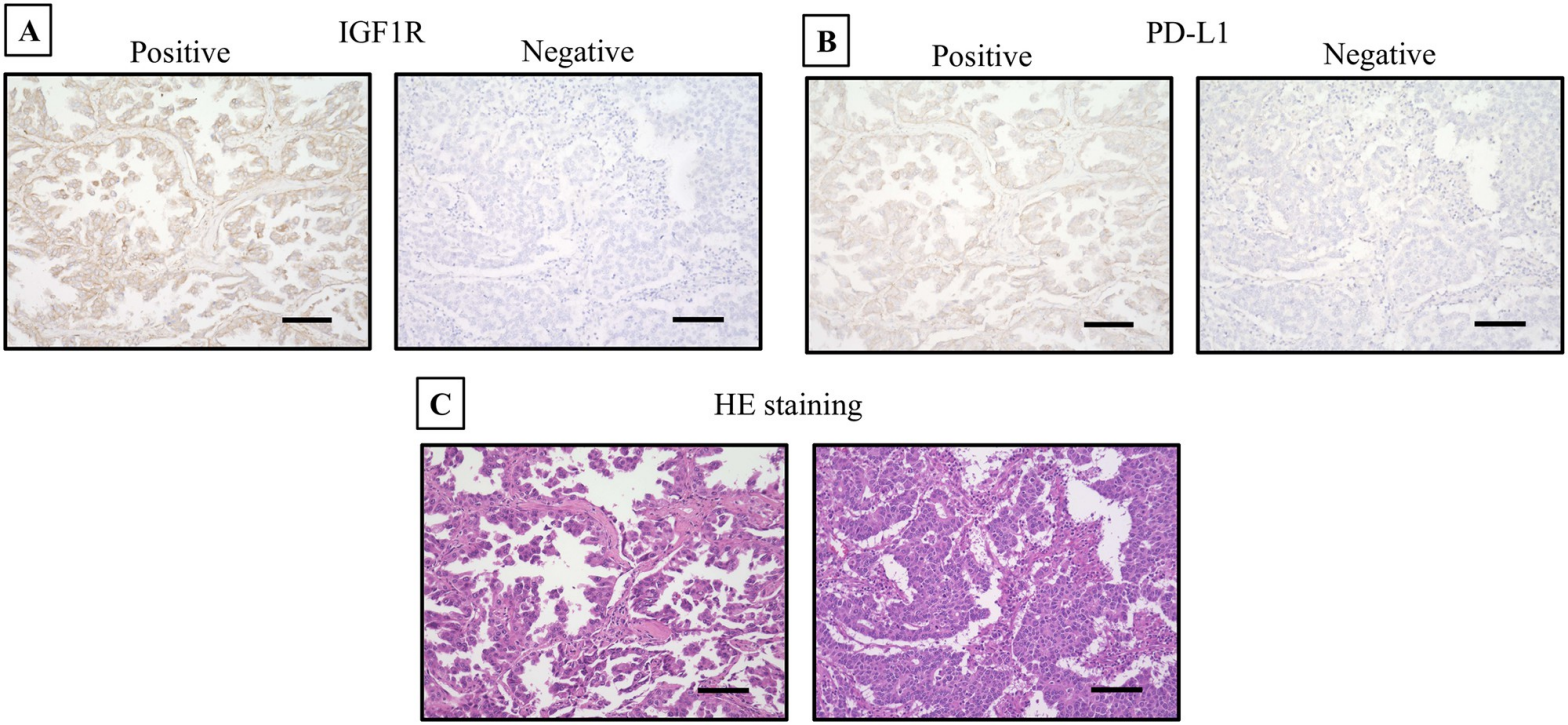
Immunostaining for IGF1R and PD-L1, and hematoxylin-eosin staining. **(A)** Immunostaining for IGF1R reveals predominant cytoplasmic staining. **(B)** Immunostaining for PD-L1 reveals predominant cytoplasmic staining. **(C)** Hematoxylin-eosin staining.

**Fig 2:**
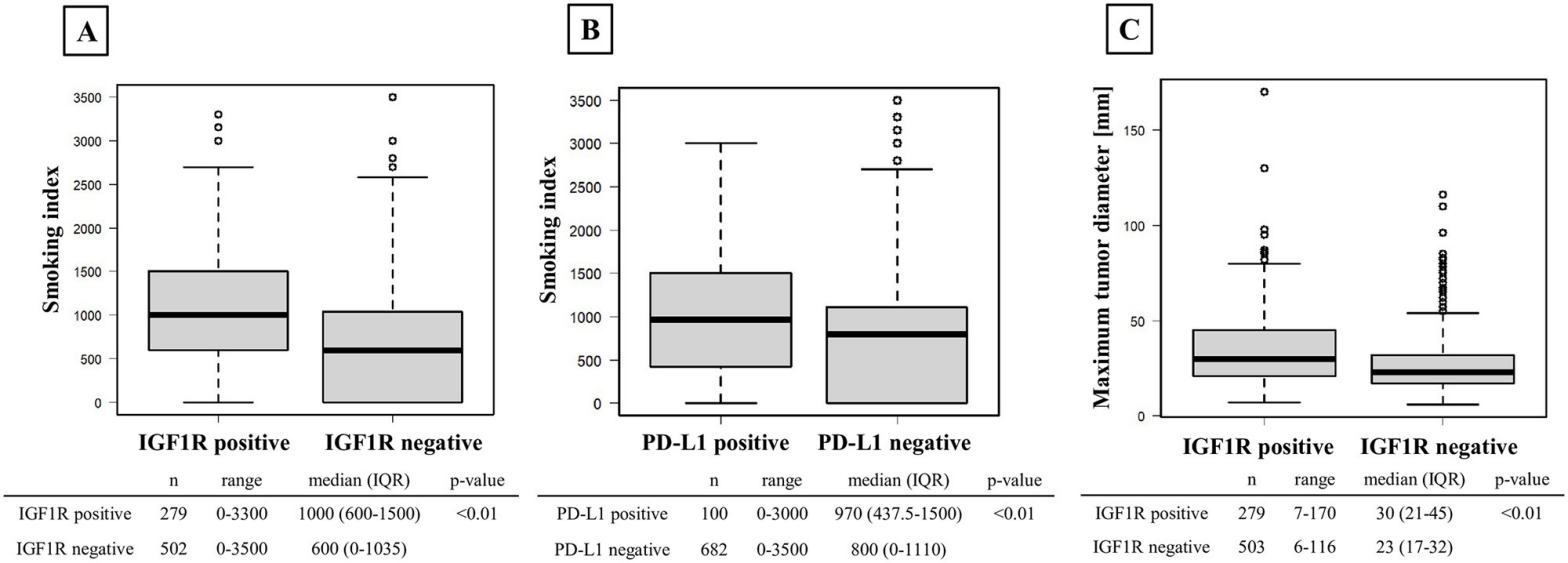
The association between IGF1R or PD-L1 and smoking index, as well as the correlation between IGF1R and tumor size. **(A)** Comparison of smoking index between the IGF1R-positive and negative groups. The IGF1R-positive group had a significantly larger smoking index. **(B)** Comparison of smoking index between PD-L1-positive and negative groups. The PD-L1-positive group had a significantly larger smoking index. **(C)** Comparison of maximum tumor diameter between IGF1R-positive and negative groups. The IGF1R-positive group had a significantly larger maximum tumor diameter.

A total of 782 patients were included in this study. Both IGF1R and PD-L1 were predominantly stained in the cytoplasm (Fig 1A–1C). IGF1R was identified as positive in 279 patients (35.8%). The median age of the participants was 70.0 years (range = 33–91), with 497 (63.6%) patients being male. Five hundred twenty-one patients had adenocarcinoma, 217 had SCC, 14 had adenosquamous cell carcinoma, 11 had large cell carcinoma, nine had pleomorphic carcinoma, three had large cell neuroendocrine carcinoma, two had adenocarcinoma in situ, one had mixed adenocarcinoma and small cell carcinoma, one had SCC and small cell carcinoma, one had NSCLC not otherwise specified, one had mucoepidermoid carcinoma, and one had atypical adenomatous hyperplasia. The IGF1R-positive group exhibited significantly higher proportions of patients who were ≥65 years old, male, had a smoking history, a performance status (PS) ≥1, had SCC, pStage≥2, exhibited pleural invasion, had vascular invasion, or showed PD-L1 expression (all p<0.01, p<0.01, p<0.01, p<0.01, p<0.01, p<0.01, p = 0.02, p<0.01, p<0.01, respectively) (Table 1). Multivariable analysis revealed that PD-L1 expression is associated with IGF1R expression (Table 2). Both IGF1R-positive and PD-L1-positive group had a significantly higher smoking index (p<0.01) (Fig 2A and 2B), and the IGF1R-positive group had a significantly larger maximum tumor diameter (p<0.01) (Fig 2C).

**Table 1:** Patient characteristics.

	No. of all patients	IGF1R positive	IGF1R negative	p-value
	n = 782	n = 279 (35.8%)	n = 503 (64.2%)	
Age				
<65	200 (25.6%)	50 (17.9%)	150 (29.8%)	<0.01
≥65	582 (74.4%)	229 (82.1%)	353 (70.2%)	
Sex				
Male	497 (63.6%)	210 (75.3%)	287 (57.1%)	<0.01
Female	285 (36.4%)	69 (24.7%)	216 (42.9%)	
Smoking history				
Current/Former	587 (75.1%)	243 (87.1%)	344 (68.4%)	<0.01
Never	195 (24.9%)	36 (12.9%)	159 (31.6%)	
ECOG PS				
0	573 (73.3%)	193 (68.8%)	380 (75.4%)	<0.01
1–3	115 (14.7%)	56 (20.2%)	59 (11.7%)	
Histology				
Sq	218 (27.9%)	137 (49.1%)	81 (16.1%)	<0.01
Non Sq	564 (72.1%)	142 (50.9%)	422 (83.9%)	
pStage				
0–1	494 (63.2%)	137 (49.1%)	357 (71.0%)	<0.01
2–3	288 (36.8%)	142 (50.9%)	146 (29.0%)	
Adjuvant therapy				
Done	192 (24.6%)	72 (25.8%)	120 (23.9%)	0.55
None	590 (75.4%)	207 (74.2%)	383 (76.1%)	
Pleural invasion				
Positive	228 (29.2%)	96 (34.4%)	132 (26.2%)	0.02
Negative	548 (70.1%)	181 (64.9%)	367 (73.0%)	
Lymphatic invasion				
Positive	212 (27.1%)	78 (28.0%)	134 (26.6%)	0.74
Negative	566 (72.4%)	200 (71.7%)	366 (72.8%)	
Vascular invasion				
Positive	169 (21.6%)	98 (35.1%)	71 (14.1%)	<0.01
Negative	609 (77.9%)	180 (64.5%)	429 (85.3%)	
PD-L1				
Positive	100 (12.8%)	68 (24.4%)	32 (6.4%)	<0.01
Negative	682 (87.2%)	211 (75.6%)	471 (93.6%)	

IGF1R, insulin-like growth factor 1 receptor; ECOG PS, Eastern Cooperative Oncology Group Performance Status; Sq, squamous cell carcinoma; PD-L1, programmed death ligand 1.

**Table 2:** Multivariate analysis of the relationship between IGF1R expression and PD-L1 expression.

Factor	Reference	OR (95%CI)	p-value
PD-L1	Negative	3.35 (1.95–5.73)	<0.01

IGF1R, insulin-like growth factor 1 receptor; OR, odds ratio; CI, confidence intervals; PD-L1, programmed death ligand 1.

### Relationship between IGF1R and relapse-free survival or overall survival

**Fig 3:**
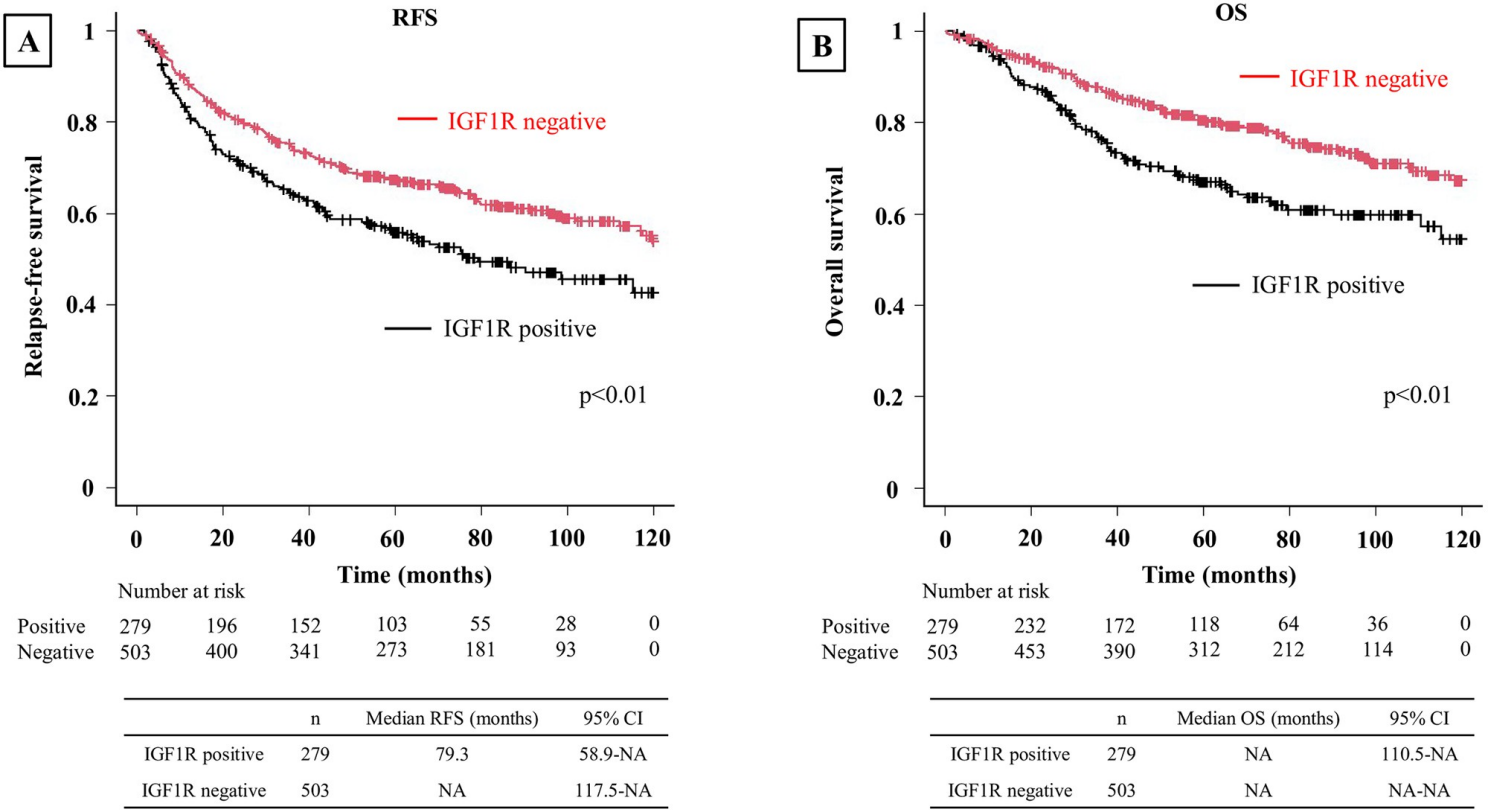
Kaplan-Meier curve depicting RFS and OS for all patients. **(A)** Kaplan-Meier curve depicting RFS for all patients. The IGF1R-positive group exhibited significantly worse RFS. **(B)** Kaplan-Meier curve depicting OS for all patients. The IGF1R-positive group exhibited significantly worse OS.

**Fig 4:**
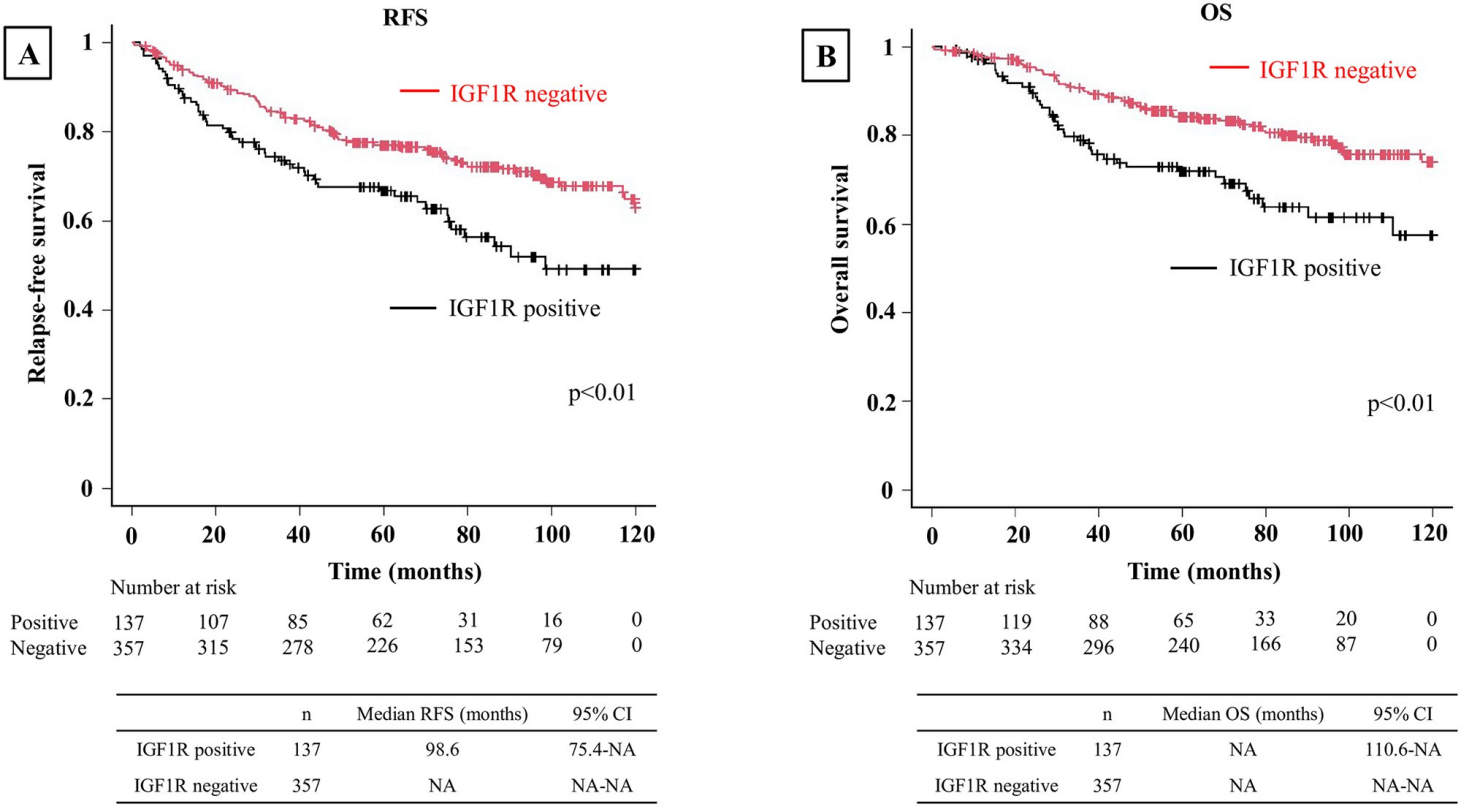
Kaplan-Meier curve depicting RFS and OS in stage 0–1 patients. **(A)** Kaplan-Meier curve depicting RFS in stage 0–1 patients. The IGF1R-positive group exhibited significantly worse RFS. **(B)** Kaplan-Meier curve depicting OS in stage 0–1 patients. The IGF1R-positive group exhibited significantly worse OS.

**Fig 5:**
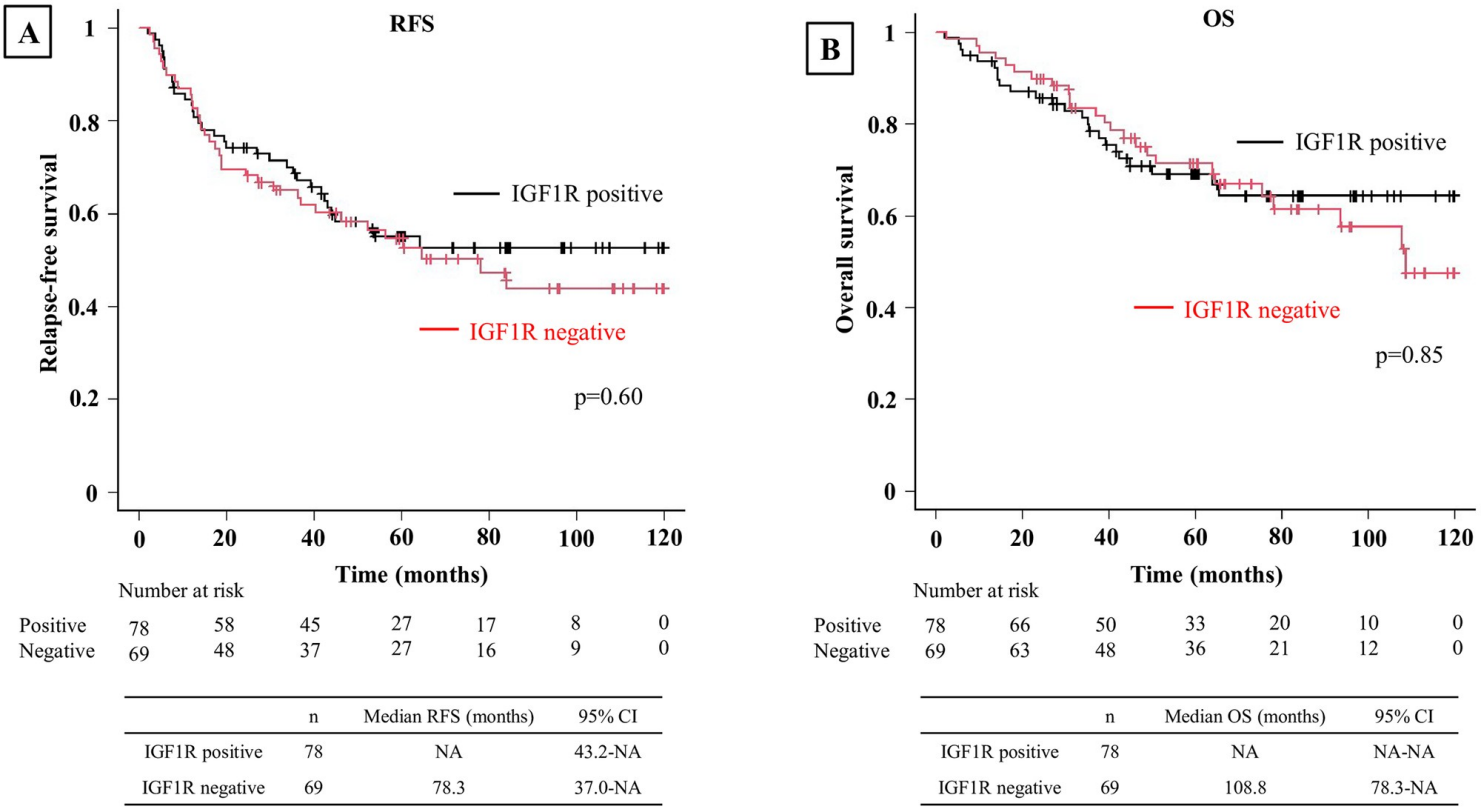
Kaplan-Meier curve depicting RFS and OS in stage 2 patients. **(A)** Kaplan-Meier curve depicting RFS in stage 2 patients. No significant difference was observed between the IGF1R-positive and negative groups. **(B)** Kaplan-Meier curve for OS in stage 2 patients. No significant difference was observed between the IGF1R-positive and negative groups.

**Fig 6:**
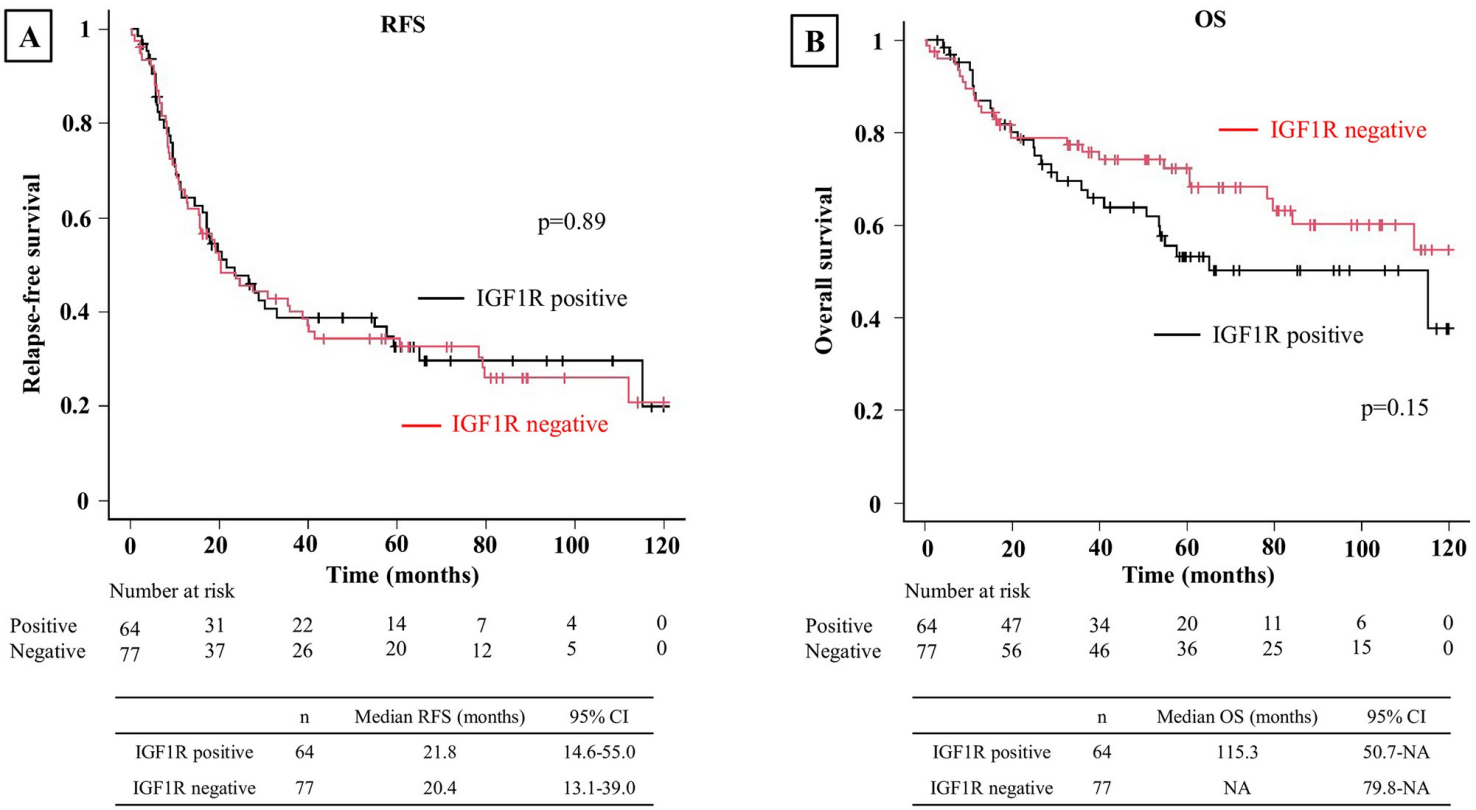
Kaplan-Meier curve depicting RFS and OS in stage 3 patients. **(A)** Kaplan-Meier curve depicting RFS in stage 3 patients. No significant difference in RFS was observed between the IGF1R-positive and negative groups. **(B)** Kaplan-Meier curve depicting OS in stage 3 patients. No significant difference was observed between the IGF1-positive and negative groups.

In all patients, IGF1R positivity was associated with worse RFS (p<0.01) and OS (p<0.01) (Fig 3A and 3B). When evaluated by stage, IGF1R positivity was associated with worse RFS (p<0.01) and OS (p<0.01) in Stage 0–1 (Fig 4A and 4B). However, no significant differences in RFS or OS were observed between IGF1R-positive and negative patients in Stage 2 (p = 0.60 and p = 0.80, respectively) (Fig 5A and 5B) or Stage 3 (p = 0.89 and p = 0.15, respectively) (Fig 6A and 6B). In univariable analysis of RFS in stage 0–1 patients, IGF1R positivity, male, smoking history, ECOG PS ≥1, pStage 2–3, pleural invasion, lymphatic invasion, and vascular invasion were all associated with significantly worse RFS (Table 3). In the multivariable analysis of RFS in stage 0–1 patients, smoking history, ECOG PS≥1, lymphatic invasion, and vascular invasion were associated with significantly worse RFS (Table 3). In univariable analysis of OS in stage 0–1 patients, IGF1R positivity, male, smoking history, ECOG PS≥1, SCC and vascular invasion were all associated with significantly worse OS (Table 4). In the multivariable analysis of OS in stage 0–1 patients, ECOG PS≥1 was associated with significantly worse OS (Table 4).

**Table 3:** Multivariable analysis of RFS in stage 0–1 patients.

Factor	Reference	RFS of stage 0–1 patients (n = 494)					
		Univariable analysis			Multivariable analysis		
		HR	95%CI	p-value	HR	95%CI	p-value
IGF1R	Negative	1.71	1.23–2.40	<0.01	1.29	0.85–1.96	0.23
Age	≤64	1.34	0.91–1.95	0.14			
Sex	Female	2.05	1.45–2.89	<0.01	1.35	0.87–2.11	0.18
Smoking history	Never	2.41	1.62–3.58	<0.01	1.75	1.04–2.95	0.04
ECOG PS	0	2.13	1.37–3.30	<0.01	2.05	1.30–3.23	<0.01
Histology	NonSq	1.52	1.05–2.21	0.03	0.73	0.45–1.18	0.20
Adjuvant therapy	Done	0.66	0.66–1.68	0.84			
Pleural invasion	Negative	1.93	1.35–2.76	<0.01	1.32	0.87–2.04	0.19
Lymphatic invasion	Negative	2.26	1.57–3.25	<0.01	1.70	1.10–2.63	0.02
Vascular invasion	Negative	2.68	1.80–3.98	<0.01	1.91	1.17–3.12	<0.01
PD-L1	Negative	1.49	0.90–2.47	0.12			

RFS, relapse-free survival; HR, hazard ratio; CI, confidence intervals; IGF1R, insulin-like growth factor 1 receptor; ECOG PS, Eastern Cooperative Oncology Group Performance Status; Sq, squamous cell carcinoma; PD-L1, programmed death ligand 1.

**Table 4:** Multivariable analysis of OS in stage 0–1 patients.

Factor	Reference	OS of stage 0–1 patients (n = 494)					
		Univariable analysis			Multivariable analysis		
		HR	95%CI	p-value	HR	95%CI	p-value
IGF1R	Negative	1.98	1.35–2.91	<0.01	1.40	0.86–2.29	0.17
Age	≤64	1.26	0.81–1.94	0.31			
Sex	Female	2.79	1.82–4.29	<0.01	1.69	0.99–2.91	0.06
Smoking history	Never	2.81	1.73–4.57	<0.01	1.70	0.90–3.21	0.10
ECOG PS	0	2.61	1.60–4.28	<0.01	2.24	1.34–3.73	<0.01
Histology	NonSq	2.10	1.39–3.16	<0.01	0.95	0.55–1.64	0.86
Adjuvant therapy	Done	1.14	0.65–1.99	0.65			
Pleural invasion	Negative	1.34	0.86–2.09	0.20			
Lymphatic invasion	Negative	1.39	0.86–2.23	0.18			
Vascular invasion	Negative	2.19	1.36–3.53	<0.01	1.52	0.85–2.70	0.15
PD-L1	Negative	1.71	0.98–3.01	0.06			

OS, overall survival; HR, hazard ratio; CI, confidence intervals; IGF1R, insulin-like growth factor 1 receptor; ECOG PS, Eastern Cooperative Oncology Group Performance Status; Sq, squamous cell carcinoma; PD-L1, programmed death ligand 1.

## Discussion

Although previous reports have not consistently linked IGF1R expression to postoperative recurrence or prognosis in lung cancer [11–14], our large-scale, long-term observational study demonstrates that IGF1R expression is associated with worse RFS and OS, particularly in the early stages. In addition, in this study, IGF1R expression was significantly correlated with PD-L1 expression, smoking history, and tumor size.

The IGF1R-positive group also showed significantly larger tumor sizes. IGF1R is a factor associated with tumor growth [4]. Upon binding with its ligands IGF1 or IGF2, IGF1R activates downstream signaling pathways such as the phosphoinositide 3-kinase (PI3K)/Akt and extracellular signal-regulated kinase (ERK) pathways, which leads to tumor cell proliferation [18–20]. This is consistent with the result of the relationship between IGF1R expression and tumor size. In addition, IGF1R expression was associated with shorter RFS and OS, particularly in early-stage lung cancer. It has been known that stage 1 has a lower frequency and amount of postoperative molecular residual disease (MRD) compared to stage 2 or 3 in lung cancer [21]. Although the immune system may eliminate minimal MRD in early-stage lung cancer, our results suggested that IGF1R expression was associated with PD-L1 expression, which might have allowed cancer cells to escape immune surveillance [22, 23] and increased the likelihood of relapse in IGF1R-positive patients. Additionally, IGF1R expression may facilitate tumor growth, leading to earlier recurrence compared to IGF1R-negative cases. Also, IGF1R expression has been known to correlate with the expression of ATP–binding cassette subfamily G member 2 (ABCG2) [24], a potential marker for cancer stem cells [25] and involved in chemoresistance [26]. These findings might explain the treatment resistance of recurrent tumors and may account for the worse OS observed in early-stage lung cancer with IGF1R positivity, where recurrence is more frequent. Many treatment strategies using ICI for neoadjuvant and adjuvant chemotherapy have been developed in recent years [27, 28]. However, due to the favorable prognosis of early-stage lung cancer, it is less amenable to the benefits of neoadjuvant and adjuvant chemotherapy. Based on our study findings, IGF1R expression may serve as an informative marker for determining appropriate perioperative chemotherapy for patients with early-stage lung cancer. Furthermore, despite promising preclinical indications, IGF1R inhibitors have not proven consistently effective in various types of cancer in clinical trials [29–31]. However, recent experiments in mice have demonstrated that the combination of an IGF1R inhibitor and an anti-PD-1 antibody synergistically inhibits tumor growth [32, 33]. IGF1R expression is correlated with the tumor microenvironment and inhibits the activation of effector cytotoxic CD8^+^ T cells [34, 35]. Therefore, inhibition of the IGF1R pathway has the potential to enhance the effectiveness of ICI by activating effector cytotoxic CD8 T cells [36]. From those results, Combining IGF1R inhibitors with ICI may hold promise for future therapies, especially as perioperative treatment for early-stage lung cancer.

In this study, we observed a positive correlation between the expression of IGF1R and PD-L1. Notably, in head and neck squamous cell carcinoma, where smoking poses a risk [37], Interleukin-6 (IL-6) has been reported to upregulate IGF1R and PD-L1 expression [16]. In the present study, the IGF1R-positive group had a significantly higher smoking index, consistent with previous studies linking IGF1R expression to smoking [14]. Smoking induces IL-6 expression in the lungs [38], potentially contributing to the upregulation of both IGF1R and PD-L1 expression in lung cancer. Additionally, the IGF1R-positive group had a significantly higher proportion of SCC, widely known to be associated with smoking [17]. Smoking-induced inflammation also enhances COX2 expression, which is associated with IGF1R expression in lung cancer [10]. Various smoking-related mechanisms, such as IL-6 and COX-2, may elucidate the association between IGF1R expression, smoking, SCC, and PD-L1 expression. However, further research is needed to elucidate the mechanism of co-expression of IGF1R and PD-L1. Our unpublished data indicate that OMUL-1, a human squamous lung cancer cell line, expresses high levels of both IGF1R and PD-L1. This cell line may be useful for analyzing the mechanisms responsible for the co-expression of IGF1R and PD-L1.

IGF1R expression was correlated with various factors, such as PD-L1, sex, and age, which differed between the IGF1R-positive and negative groups. Additionally, IGF1R expression was positively correlated with smoking index. Given the high prevalence of male smokers in Japan [39], the prevalence of IGF1R-positive lung cancer may be higher in males. Moreover, the smoking index tends to be higher in older patients, and the population of nonsmokers has decreased in recent years [39], which may explain the large number of older adults in the IGF1R-positive group.

One limitation of this study stems from its retrospective design. Further research and prospective study are necessary to fully understand the clinical implications of IGF1R expression. Furthermore, prospective clinical trials on the combination therapy of IGF1R inhibitors and ICIs are warranted. The relationship between IGF1R expression and the effects of ICI was previously unknown and requires further study. In addition, future research should investigate whether IGF1R expression is related to immune cell infiltration and elucidate the underlying mechanisms.

In conclusion, our study reveals that higher IGF1R expression correlates with poorer outcomes in terms of RFS and OS, particularly in patients with early-stage lung cancer. Furthermore, our findings indicate a relationship between IGF1R expression in lung cancer and several factors: PD-L1 expression, smoking history, and tumor size.

## Supporting information

S1 Data (XLSX)

## References

[1] Wu J, Yu E. Insulin-like growth factor receptor-1 (IGF-IR) as a target for prostate cancer therapy. Cancer Metastasis Rev. 2014;33(2–3):607–17. doi: 10.1007/s10555-013-9482-0 . 24414227 PMC4096322

[2] Motallebnezhad M, Aghebati-Maleki L, Jadidi-Niaragh F, Nickho H, Samadi-Kafil H, Shamsasenjan K, et al. The insulin-like growth factor-I receptor (IGF-IR) in breast cancer: biology and treatment strategies. Tumour Biol. 2016;37(9):11711–21. Epub 20160721. doi: 10.1007/s13277-016-5176-x . 27444280

[3] Hu L, Xu X, Li Q, Chen X, Yuan X, Qiu S, et al. Caveolin-1 increases glycolysis in pancreatic cancer cells and triggers cachectic states. Faseb j. 2021;35(8):e21826. doi: 10.1096/fj.202100121RRR . 34320244

[4] Heidegger I, Kern J, Ofer P, Klocker H, Massoner P. Oncogenic functions of IGF1R and INSR in prostate cancer include enhanced tumor growth, cell migration and angiogenesis. Oncotarget. 2014;5(9):2723–35. doi: 10.18632/oncotarget.1884 . 24809298 PMC4058040

[5] Xu XL, Guo AX, Pan QY, Chang AL, Zhao CR. MiR-99a suppresses cell migration and invasion by regulating IGF1R in gastric cancer. Eur Rev Med Pharmacol Sci. 2019;23(17):7375–82. doi: 10.26355/eurrev_201909_18845 . 31539124

[6] Alfaro-Arnedo E, López IP, Piñeiro-Hermida S, Canalejo M, Gotera C, Sola JJ, et al. IGF1R acts as a cancer-promoting factor in the tumor microenvironment facilitating lung metastasis implantation and progression. Oncogene. 2022;41(28):3625–39. Epub 20220610. doi: 10.1038/s41388-022-02376-w . 35688943 PMC9184253

[7] Joehlin-Price AS, Stephens JA, Zhang J, Backes FJ, Cohn DE, Suarez AA. Endometrial Cancer Insulin-Like Growth Factor 1 Receptor (IGF1R) Expression Increases with Body Mass Index and Is Associated with Pathologic Extent and Prognosis. Cancer Epidemiol Biomarkers Prev. 2016;25(3):438–45. Epub 20151218. doi: 10.1158/1055-9965.EPI-15-1145 . 26682991 PMC5075967

[8] Du P, Liu F, Liu Y, Shao M, Li X, Qin G. Linc00210 enhances the malignancy of thyroid cancer cells by modulating miR-195-5p/IGF1R/Akt axis. J Cell Physiol. 2020;235(2):1001–12. Epub 20190625. doi: 10.1002/jcp.29016 . 31240707

[9] Xu X, Qiu Y, Chen S, Wang S, Yang R, Liu B, et al. Different Roles of the Insulin-like Growth Factor (IGF) Axis in Non-small Cell Lung Cancer. Curr Pharm Des. 2022;28(25):2052–64. doi: 10.2174/1381612828666220608122934 . 36062855

[10] Põld M, Krysan K, Põld A, Dohadwala M, Heuze-Vourc’h N, Mao JT, et al. Cyclooxygenase-2 modulates the insulin-like growth factor axis in non-small-cell lung cancer. Cancer Res. 2004;64(18):6549–55. doi: 10.1158/0008-5472.CAN-04-1225 . 15374967

[11] Dziadziuszko R, Merrick DT, Witta SE, Mendoza AD, Szostakiewicz B, Szymanowska A, et al. Insulin-like growth factor receptor 1 (IGF1R) gene copy number is associated with survival in operable non-small-cell lung cancer: a comparison between IGF1R fluorescent in situ hybridization, protein expression, and mRNA expression. J Clin Oncol. 2010;28(13):2174–80. Epub 20100329. doi: 10.1200/JCO.2009.24.6611 . 20351332 PMC2860435

[12] Gately K, Forde L, Cuffe S, Cummins R, Kay EW, Feuerhake F, et al. High coexpression of both EGFR and IGF1R correlates with poor patient prognosis in resected non-small-cell lung cancer. Clin Lung Cancer. 2014;15(1):58–66. Epub 20131107. doi: 10.1016/j.cllc.2013.08.005 . 24210543

[13] Nakagawa M, Uramoto H, Oka S, Chikaishi Y, Iwanami T, Shimokawa H, et al. Clinical significance of IGF1R expression in non-small-cell lung cancer. Clin Lung Cancer. 2012;13(2):136–42. Epub 20111201. doi: 10.1016/j.cllc.2011.10.006 . 22133293

[14] Zhao S, Qiu Z, He J, Li L, Li W. Insulin-like growth factor receptor 1 (IGF1R) expression and survival in non-small cell lung cancer patients: a meta-analysis. Int J Clin Exp Pathol. 2014;7(10):6694–704. Epub 20140915. . 25400749 PMC4230063

[15] Yu H, Boyle TA, Zhou C, Rimm DL, Hirsch FR. PD-L1 Expression in Lung Cancer. J Thorac Oncol. 2016;11(7):964–75. Epub 20160423. doi: 10.1016/j.jtho.2016.04.014 . 27117833 PMC5353357

[16] Li J, Xiao Y, Yu H, Jin X, Fan S, Liu W. Mutual connected IL-6, EGFR and LIN28/Let7-related mechanisms modulate PD-L1 and IGF upregulation in HNSCC using immunotherapy. Front Oncol. 2023;13:1140133. Epub 20230412. doi: 10.3389/fonc.2023.1140133 . 37124491 PMC10130400

[17] Wang X, Wang T, Hua J, Cai M, Qian Z, Wang C, et al. Histological types of lung cancer attributable to fine particulate, smoking, and genetic susceptibility. Sci Total Environ. 2023;858(Pt 2):159890. Epub 20221102. doi: 10.1016/j.scitotenv.2022.159890 . 36334679

[18] Kasprzak A, Kwasniewski W, Adamek A, Gozdzicka-Jozefiak A. Insulin-like growth factor (IGF) axis in cancerogenesis. Mutat Res Rev Mutat Res. 2017;772:78–104. Epub 20160904. doi: 10.1016/j.mrrev.2016.08.007 . 28528692

[19] Stefani C, Miricescu D, Stanescu S II, Nica RI, Greabu M, Totan AR, et al. Growth Factors, PI3K/AKT/mTOR and MAPK Signaling Pathways in Colorectal Cancer Pathogenesis: Where Are We Now? Int J Mol Sci. 2021;22(19). Epub 20210923. doi: 10.3390/ijms221910260 . 34638601 PMC8508474

[20] Werner H. The IGF1 Signaling Pathway: From Basic Concepts to Therapeutic Opportunities. Int J Mol Sci. 2023;24(19). Epub 20231004. doi: 10.3390/ijms241914882 . 37834331 PMC10573540

[21] Chaudhuri AA, Chabon JJ, Lovejoy AF, Newman AM, Stehr H, Azad TD, et al. Early Detection of Molecular Residual Disease in Localized Lung Cancer by Circulating Tumor DNA Profiling. Cancer Discov. 2017;7(12):1394–403. Epub 20170924. doi: 10.1158/2159-8290.CD-17-0716 . 28899864 PMC5895851

[22] Chen DS, Mellman I. Oncology meets immunology: the cancer-immunity cycle. Immunity. 2013;39(1):1–10. doi: 10.1016/j.immuni.2013.07.012 . 23890059

[23] Chen J, Jiang CC, Jin L, Zhang XD. Regulation of PD-L1: a novel role of pro-survival signalling in cancer. Ann Oncol. 2016;27(3):409–16. Epub 20151217. doi: 10.1093/annonc/mdv615 . 26681673

[24] Kim CK, Oh S, Kim SJ, Leem SH, Heo J, Chung SH. Correlation of IGF1R expression with ABCG2 and CD44 expressions in human osteosarcoma. Genes Genomics. 2018;40(4):381–8. Epub 20171201. doi: 10.1007/s13258-017-0639-z . 29892839

[25] Ding XW, Wu JH, Jiang CP. ABCG2: a potential marker of stem cells and novel target in stem cell and cancer therapy. Life Sci. 2010;86(17–18):631–7. Epub 20100214. doi: 10.1016/j.lfs.2010.02.012 . 20159023

[26] Ejendal KF, Hrycyna CA. Multidrug resistance and cancer: the role of the human ABC transporter ABCG2. Curr Protein Pept Sci. 2002;3(5):503–11. doi: 10.2174/1389203023380521 . 12369998

[27] Forde PM, Spicer J, Lu S, Provencio M, Mitsudomi T, Awad MM, et al. Neoadjuvant Nivolumab plus Chemotherapy in Resectable Lung Cancer. N Engl J Med. 2022;386(21):1973–85. Epub 20220411. doi: 10.1056/NEJMoa2202170 . 35403841 PMC9844511

[28] Felip E, Altorki N, Zhou C, Csőszi T, Vynnychenko I, Goloborodko O, et al. Adjuvant atezolizumab after adjuvant chemotherapy in resected stage IB-IIIA non-small-cell lung cancer (IMpower010): a randomised, multicentre, open-label, phase 3 trial. Lancet. 2021;398(10308):1344–57. Epub 20210920. doi: 10.1016/S0140-6736(21)02098-5 . 34555333

[29] Qu X, Wu Z, Dong W, Zhang T, Wang L, Pang Z, et al. Update of IGF-1 receptor inhibitor (ganitumab, dalotuzumab, cixutumumab, teprotumumab and figitumumab) effects on cancer therapy. Oncotarget. 2017;8(17):29501–18. doi: 10.18632/oncotarget.15704 . 28427155 PMC5438747

[30] Chiappori AA, Otterson GA, Dowlati A, Traynor AM, Horn L, Owonikoko TK, et al. A Randomized Phase II Study of Linsitinib (OSI-906) Versus Topotecan in Patients With Relapsed Small-Cell Lung Cancer. Oncologist. 2016;21(10):1163–4. Epub 20160930. doi: 10.1634/theoncologist.2016-0220 . 27694157 PMC5061534

[31] Fassnacht M, Berruti A, Baudin E, Demeure MJ, Gilbert J, Haak H, et al. Linsitinib (OSI-906) versus placebo for patients with locally advanced or metastatic adrenocortical carcinoma: a double-blind, randomised, phase 3 study. Lancet Oncol. 2015;16(4):426–35. Epub 20150318. doi: 10.1016/S1470-2045(15)70081-1 . 25795408

[32] Ajona D, Ortiz-Espinosa S, Lozano T, Exposito F, Calvo A, Valencia K, et al. Short-term starvation reduces IGF-1 levels to sensitize lung tumors to PD-1 immune checkpoint blockade. Nat Cancer. 2020;1(1):75–85. Epub 20200113. doi: 10.1038/s43018-019-0007-9 . 35121837

[33] Kang B, Zhang X, Wang W, She S, Chen W, Chen C, et al. The Novel IGF-1R Inhibitor PB-020 Acts Synergistically with Anti-PD-1 and Mebendazole against Colorectal Cancer. Cancers (Basel). 2022;14(23). Epub 20221123. doi: 10.3390/cancers14235747 . 36497233 PMC9737525

[34] Galifi CA, Wood TL. Insulin-like growth factor-1 receptor crosstalk with integrins, cadherins, and the tumor microenvironment: sticking points in understanding IGF1R function in cancer. Endocr Relat Cancer. 2023;30(10). Epub 20230825. doi: 10.1530/erc-23-0031 . 37490874 PMC13418071

[35] Pellegrino M, Secli V, D’Amico S, Petrilli LL, Caforio M, Folgiero V, et al. Manipulating the tumor immune microenvironment to improve cancer immunotherapy: IGF1R, a promising target. Front Immunol. 2024;15:1356321. Epub 20240214. doi: 10.3389/fimmu.2024.1356321 . 38420122 PMC10899349

[36] Raskov H, Orhan A, Christensen JP, Gögenur I. Cytotoxic CD8(+) T cells in cancer and cancer immunotherapy. Br J Cancer. 2021;124(2):359–67. Epub 20200915. doi: 10.1038/s41416-020-01048-4 . 32929195 PMC7853123

[37] Hayes RB, Ahn J, Fan X, Peters BA, Ma Y, Yang L, et al. Association of Oral Microbiome With Risk for Incident Head and Neck Squamous Cell Cancer. JAMA Oncol. 2018;4(3):358–65. doi: 10.1001/jamaoncol.2017.4777 . 29327043 PMC5885828

[38] Francus T, Romano PM, Manzo G, Fonacier L, Arango N, Szabo P. IL-1, IL-6, and PDGF mRNA expression in alveolar cells following stimulation with a tobacco-derived antigen. Cell Immunol. 1992;145(1):156–74. doi: 10.1016/0008-8749(92)90320-o . 1423641

[39] Tanaka H, Mackenbach JP, Kobayashi Y. Widening Socioeconomic Inequalities in Smoking in Japan, 2001–2016. J Epidemiol. 2021;31(6):369–77. Epub 20201125. doi: 10.2188/jea.JE20200025 . 32595181 PMC8126678

